# Leveraging the 72-Hour Rule Change to Support Transition From Hospital to Opioid Treatment Program

**DOI:** 10.1001/jamanetworkopen.2025.44996

**Published:** 2025-11-21

**Authors:** Susan L. Calcaterra, Melissa B. Weimer, Eric Grimm, Yevgeniya Scherbak, Rawan Abdel Galil, Olivia Berger, Lindsay A. Bowman, Suzanne A. Nesbit, Alexandra Barany, Megan E. Buresh

**Affiliations:** 1 Department of Medicine, Division of Hospital Medicine, University of Colorado, Aurora; 2 Department of Medicine, Division of General Internal Medicine, University of Colorado, Aurora; 3 Department of Medicine, Yale School of Medicine, New Haven, Connecticut; 4 Department of Chronic Disease Epidemiology, Yale School of Public Health, New Haven, Connecticut; 5 UCHealth, University of Colorado Hospital, Aurora; 6 Skaggs School of Pharmacy and Pharmaceutical Sciences, University of Colorado, Aurora; 7 Division of Addiction Medicine, Johns Hopkins School of Medicine, Baltimore, Maryland; 8 Department of Pharmacy, Johns Hopkins Bayview Medical Center, Baltimore, Maryland; 9 Department of Pharmacy, The Johns Hopkins Hospital, Baltimore, Maryland; 10 Office of Graduate Clinical Education, Johns Hopkins School of Medicine; 11 Department of Epidemiology, Johns Hopkins School of Public Health, Baltimore, Maryland

## Abstract

**Importance:**

Changes have been made to the US federal 72-hour rule, which allows hospitals to administer or dispense but not prescribe up to 72 hours’ worth of methadone for opioid withdrawal or opioid use disorder (OUD) at discharge (ie, take-home doses).

**Objective:**

To characterize acute care encounters involving take-home methadone and identify variables associated with completion of the transition from hospital to opioid treatment program (OTP) within 72 hours (hereafter 72-hour hospital to OTP linkage).

**Design, Setting, and Participants:**

This cohort study examined hospital and emergency department encounters for patients with OUD who received take-home methadone doses from 4 geographically diverse hospitals between November 2022 and April 2024.

**Exposure:**

Receipt of take-home methadone at hospital discharge.

**Main Outcomes and Measures:**

The primary outcome was documentation of 72-hour hospital to OTP linkage. Multinomial logistic regression was used to analyze the association between OTP linkage status (yes, no, or unknown) and discharge disposition, OTP enrollment at the time of the acute care encounter, co-use of opioids and stimulants, methadone dose, pregnancy, length of hospital stay, and psychiatric conditions.

**Results:**

Included in the analysis were 519 encounters for 456 individuals (median [IQR] age, 46 [36-56] years) who received take-home methadone at hospital discharge. Of these encounters, 280 (53.9%) involved patients who were male. Weekend discharge was the most common reason for dispensing take-home methadone doses (342 encounters [65.9%]). Overall, 231 encounters (44.5%) involved patients who completed a 72-hour hospital-to-OTP linkage, 67 encounters (12.9%) did not link to an OTP within 72 hours of discharge, and 221 (42.6%) had an unknown 72-hour OTP linkage status. Variables associated with completion of 72-hour hospital to OTP linkage included discharge to a postacute care facility (odds ratio [OR], 2.12; 95% CI, 1.12-4.21; *P* = .02), OTP enrollment at the time of the acute care encounter (OR, 2.63; 95% CI, 1.48-4.79; *P* < .001), and increasing methadone dose in 10-mg increments (OR, 1.20; 95% CI, 1.09-1.33; *P* < .001). Co-use of opioids and stimulants was inversely associated with completion of the 72-hour hospital to OTP linkage (OR, 0.48; 95% CI, 0.26-0.87; *P* = .01).

**Conclusions and Relevance:**

In this cohort study, preexisting relationships between hospitals, postacute care facilities, and OTPs, along with provision of take-home methadone, likely facilitated OTP linkage. Expansion of these relationships and processes could facilitate OUD treatment engagement during tenuous care transitions.

## Key Points

**Question:**

What variables are associated with transition from hospital to opioid treatment program (OTP) under the revised 72-hour rule, which allows methadone doses for opioid use disorder (OUD) to be dispensed to patients at hospital discharge?

**Findings:**

In this cohort study examining 519 acute care encounters for 456 individuals who received take-home methadone doses at hospital discharge, discharge to postacute care facilities, current OTP enrollment, and higher methadone doses were directly associated with the transition from hospital to OTP within 72 hours.

**Meaning:**

Findings of this study suggest that preexisting relationships among hospitals, postacute care facilities, and OTPs along with provision of methadone at hospital discharge likely facilitated the transition to OTP; expansion of these relationships and processes could facilitate OUD treatment engagement during tenuous care transitions.

## Teaser

This cohort study assesses the variables associated with the hospital to opioid treatment program transition under changes to the 72-hour rule, which permits 3 take-home doses of methadone to be dispensed at hospital discharge for opioid use disorder.

## Introduction

In an era of lethal, potent synthetic opioids, hospitals have become an essential site of care for people with opioid use disorder (OUD).^1,2,3,4^ Methadone is a first-line medication to treat OUD and protects against overdose deaths.^5,6,7,8^ In the US there are no federal restrictions on methadone use to manage opioid withdrawal symptoms when patients are hospitalized with a medical or surgical condition other than OUD.^9,10^

In the outpatient setting, methadone for OUD is regulated by federal and state governments and can be legally dispensed only from federally accredited and certified opioid treatment programs ([OTPs] ie, methadone clinics).^11,12^ Buprenorphine for OUD treatment can be prescribed by US Drug Enforcement Administration–licensed clinicians and a 30-day supply can be dispensed from an outpatient pharmacy. In contrast, when methadone is initiated in the hospital, the patient must follow up at an OTP within approximately 24 hours of hospital discharge to receive their next methadone dose to avoid experiencing uncomfortable opioid withdrawal symptoms.^11,13,14,15^ Barriers to the transition from hospital to OTP (hereafter hospital to OTP linkage) include opioid withdrawal and pain; lack of transportation, proof of identification, and housing; and unstable medical or mental illness.^16,17^ Due to the lethality of the unregulated opioid supply, transitional periods, including the time from hospital discharge to follow-up at an OTP, can be a precarious time for patients with OUD.^18^ Such transitional periods are associated with an increased risk of overdose^19,20^ and death.^21,22^

In August 2023, US Drug Enforcement Administration Title 21, Chapter II, Part 1306.07(b) of the Code of Federal Regulations (hereafter the 72-hour rule) was amended to allow practitioners to dispense (but not prescribe) up to a 3-day supply of take-home methadone for opioid withdrawal at one time “for the purpose of initiating maintenance treatment or detoxification treatment (or both).”^9,23^ The regulation previously permitted narcotics to be administered daily up to 3 days for emergency treatment and to prevent opioid withdrawal.^14^ In response to this change, some hospital-based addiction consultation services (ACSs) developed protocols using an order process within the electronic health record to dispense a bridge supply of methadone to patients with OUD at hospital discharge. These processes intend to facilitate hospital to OTP linkage during care transitions, especially when patients are discharged from the hospital outside of OTP business hours or from the hospital to postacute care settings.^15,24,25^

This study aimed to identify encounter and patient characteristics associated with 72-hour hospital to OTP linkage following receipt of take-home methadone at hospital discharge. We hypothesized that discharge disposition to postacute care facilities and higher methadone doses would be associated with OTP linkage.

## Methods

### Study Design and Setting

This multisite retrospective cohort study involved hospital and emergency department (ED) encounter data from Yale New Haven Hospital (YNHH), Johns Hopkins Hospital (JHH), Johns Hopkins Bayview Medical Center (JHBMC), and University of Colorado Hospital (UCH) between November 2022 to April 2024. All 4 hospitals supported ACSs during the study period. ACS team members, inpatient pharmacists, and other key partners at each hospital collaborated to implement federally compliant processes whereby methadone was dispensed from the inpatient pharmacy to patients with OUD prior to discharge.^24,26^ In accordance with federal regulations, patients who received take-home methadone were referred to local OTPs.^14^ JHH and JHBMC implemented their take-home methadone process in the inpatient and ED settings in November 2022; YNHH and UCH implemented their processes in the inpatient setting in April 2023. This study was approved by each site’s institutional review board, informed consent was waived due to the retrospective nature of the study, and data use agreements were obtained for data sharing. This study is reported in accordance with the Strengthening the Reporting of Observational Studies in Epidemiology (STROBE) reporting guideline for cohort studies.

### Data Abstraction

A data abstraction form was developed using an Excel file (version 2024; Microsoft) with prespecified variables of interest. Data input into the file were fixed as yes or no or drop-down choices, including discharge department, hospital location (Connecticut, Maryland, or Colorado), encounter type (inpatient or ED), discharge day, and so on, to standardize data and for ease of data merging and analysis. We piloted the data extraction tool, convened to review the findings, revised the abstraction tool for clarity, and finalized the abstraction tool. M.B.W. extracted data from YNHH, M.B. and R.A.G. extracted data from JHH and JHBMC, and S.L.C. extracted data from UCH. S.L.C., M.B., and M.B.W. provided clinical care within their institutions and were familiar with their hospital’s electronic health record (EHR) and where to find the requested data within it.

### Inclusion Criteria

We included encounters in which patients received at least 1 take-home methadone dose and met criteria for moderate to severe OUD. We identified encounters electronically using medication orders for take-home methadone in each hospital’s EHR. Some patients with encounters involving take-home methadone were enrolled in an OTP prior to hospitalization; others were newly initiated on methadone during hospitalization. Patients who were prescribed methadone for a pain diagnosis without an OUD diagnosis were excluded.

### Outcome

The primary outcome was documentation of a completed 72-hour hospital to OTP linkage (yes, no, or unknown). A linkage status of yes was determined by (1) direct contact with the partnering OTP to confirm whether methadone was delivered to a skilled nursing facility (SNF) or if the patient presented to the OTP (JHH and JHBMC), (2) manual medical record review of the affiliated OTP’s EHR (UCH and YNNH), and/or (3) pharmacist verification of methadone dosing via a telephone call to the patient’s OTP during a subsequent acute care encounter (UCH and YNNH).

Partner OTPs had working relationships with the sites’ ACSs to facilitate hospital to OTP linkage or to facilitate methadone deliveries to SNFs. Some affiliated OTPs shared an EHR with the hospital. In all 3 states, partnering OTPs, affiliated OTPs, and nonaffiliated OTPs had limited Saturday hours or were closed to new patients treated with methadone on weekends. No OTPs in these states were open on Sundays. Due to federal confidentiality rules applying to substance use disorder treatment outlined in 42 CFR Part 2,^27^ linkage data to nonpartner and nonaffiliated OTPs were unable to be obtained without written patient consent and were categorized as having an unknown linkage status^28^ (Table 1).

**Table 1.** Terms, Definitions, and US Federal Regulations Governing Methadone Prescribing, Administration, and Dispensing for OUD Across Healthcare Settings

Definition	Regulations
Prescribe: A prescriber writes a prescription that a patient can take to a pharmacy to be filled	Legally, a DEA-registered prescriber can write a methadone prescription for a pain indication and send it to an outpatient pharmacy When prescribed for pain, methadone is reported to the state’s PDMP Legally, a DEA-registered prescriber cannot prescribe methadone for OUD, nor can it be sent to an outpatient pharmacy
Administer: A health care provider gives a single dose directly to a patient (eg, observed ingestion or an injection)	Legally, hospitals, emergency departments, and some clinics (using an inpatient pharmacy) may administer methadone to prevent opioid withdrawal using the 72-hour rule
Dispense: The process of preparing and providing medication for a patient to take away as a take-home	Legally, OTPs are the only outpatient settings allowed to dispense methadone for OUD treatment Legally, hospitals, emergency departments, and some clinics may dispense or prepare doses for take-home use (using the inpatient pharmacy) for up to 72 h while arranging OTP follow-up Although there are some exceptions, usually methadone dispensed for OUD is not reported to the state’s PDMP
**Ability to dispense or administer methadone for OUD by care setting**	
Primary care clinic (non-OTP)	No, not for methadone maintenance therapy for OUD^11^^,^^a^
Emergency departments	Yes, they may dispense methadone by using a hospital pharmacy under the 72-hour rule while arranging referrals to OTPs^9,15^
Opioid treatment programs	Yes, OTPs may dispense or administer methadone maintenance therapy for OUD^11^ from an onsite dispensing window.^b^
General hospitals and LTACHs	Yes, they may initiate and/or continue methadone administered or dispensed during the inpatient stay when the patient is admitted for another condition (hospital exception)^9^ May dispense under the 72-hour rule at hospital discharge^9^
SNF^28^	Generally, there is no dispensing past 72 h unless the SNF is registered as an OTP or has an on-site methadone dispensing unit under an OTP^9,11^ Yes, they may store and administer methadone provided by a patient’s OTP (eg, via visiting nurse or guest dosing)^11^
Substance use treatment settings (non-OTP programs)	Generally, there is no independent OTP-style dispensing^11^ Yes, they may store and administer methadone provided by a patient’s OTP (eg, via visiting nurse or guest dosing)^11^
Long-term care nursing homes^28^	Generally, there is no independent OTP-style dispensing^11^ Yes, they may store and administer methadone provided by a patient’s OTP (eg, via visiting nurse or guest dosing) when the patient is enrolled in an OTP^11^ Mobile OTP units can deliver methadone to nursing facilities where permitted when the patient is enrolled in an OTP^11^
Correctional facilities	Yes, may initiate methadone treatment if registered with the DEA as a hospital or clinic^9,11^ If not registered with the DEA as a hospital, then the facility may dispense under the 72-hour rule while they plan for transfer or make arrangements for continued treatment^9^ Yes, may store and administer methadone provided by a patient’s OTP (eg, via visiting nurse or guest dosing)^11^

Abbreviations: DEA, Drug Enforcement Administration; LTACH, long-term acute care hospital; OTP, opioid treatment program; OUD, opioid use disorder; PDMP, prescription drug monitoring program; SNF, skilled nursing facility.

^a^ Some sites developed processes to dispense methadone in clinics by using a hospital pharmacy under the 72-hour rule while arranging referrals to OTPs.^9,26^

^b^ Methadone is dispensed in liquid formulation only.

### Variables

Variables were extracted both electronically and manually from the EHR and included patient-reported sex; age; race; ethnicity; insurance coverage; hospital location; discharge department; and discharge day, month, and year. Race and ethnicity were included as variables in the initial model to assess for associations to the outcome of interest. Race categories included American Indian or Alaska Native, Asian, Black, Native Hawaiian or Pacific Islander, and White. The American Indian or Alaska Native, Asian, and Native Hawaiian or Pacific Islander categories were collapsed into a single category due to small numbers in each group. Ethnicity was categorized as either Hispanic or non-Hispanic. We categorized discharge disposition into 3 groups: (1) leaving prior to hospital treatment completion or discharged to jail, (2) discharged to the street (ie, unhoused), to a shelter, or to home, and (3) discharged to a long-term acute care hospital (LTACH), a SNF, or acute physical rehabilitation. We manually reviewed discharge orders to identify the presence of a naloxone prescription (yes or no), and we reviewed inpatient orders to identify the number of methadone doses dispensed and milligrams dispensed. Methadone dose was analyzed as a continuous variable. We manually reviewed clinical notes to identify the reason for dispensing take-home methadone categorized as (1) holiday, (2) SNF discharge, (3) weekend discharge, or (4) other, which was most frequently due to travel distance. We identified OTP enrollment status (yes or no) at the time of the acute care encounter by EHR review. We reviewed hospital length of stay by identifying admission and discharge dates. We identified encounters involving unhealthy use of a substance or substance use disorder and those related to alcohol, stimulants, benzodiazepines, and/or drug poisoning or overdose (not mutually exclusive); pregnancy, infections (eg, skin and soft tissue infections, musculoskeletal infections, endocarditis, and bacteremia), trauma or burn, and/or psychiatric conditions (yes or no) by manually reviewing clinical notes, diagnoses on problem lists, and discharge summaries. We further categorized encounters involving psychiatric conditions such as (1) attention deficit hyperactivity disorder, (2) anxiety or depressive disorder, (3) bipolar disorder, (4) posttraumatic stress disorder, (5) schizophrenia, and (6) other psychotic conditions.

### Statistical Analysis

All data were merged, and data analysis occurred at the University of Colorado. We calculated descriptive statistics using frequencies and percentages for categorical variables and means and medians for continuous variables. We calculated 72-hour OTP linkage status as a percentage. Informed by the literature and our clinical expertise, we identified a priori the independent variables that may be related to 72-hour OTP linkage: discharge disposition; methadone dose in milligrams^29,30,31^; OTP enrollment status at hospital admission^32,33^; hospital length of stay; and encounters involving stimulants,^34,35^ psychiatric conditions,^36,37,38^ or pregnancy.^39,40^

We used multinomial logistic regression to analyze the association between the aforementioned variables and hospital to OTP linkage status (yes, no, or unknown) while controlling for age, race, ethnicity, and sex. Statistical significance was assessed using 2-sided tests with a significance level set at *P* < .05. Ninety-five percent CIs were reported for all parameter estimates. We analyzed data using SAS, version 9.4 and SAS Enterprise Guide, version 8.2 (SAS Institute Inc).

## Results

### Hospital Encounter Characteristics

Included in the analysis were 519 encounters for 456 individuals (median [IQR] age, 46 [36-56] years) who received take-home methadone. Twelve percent of the encounters represented repeated acute care encounters by individual patients. Of these encounters, 280 (53.9%) involved males and 239 (46.1%) involved females (Table 2). Fourteen encounters (2.7%) involved individuals who identified as Asian, American Indian or Alaska Native, Native Hawaiian or Pacific Islander; 167 encounters (32.2%) were for Black individuals; 337 (64.9%) involved White individuals; and 1 (0.2%) involved a multiracial individual. A total of 488 encounters (94.0%) involved non-Hispanic individuals and 31 (6.0%) involved Hispanic individuals. Most encounters (401 [77.3%]) listed Medicaid as the primary insurance and occurred in Maryland (407 [78.4%]). The most frequently listed discharge department on the encounters was medicine or a medicine subspeciality (355 [68.4%]) followed by surgery or a surgical subspeciality (88 [17%]), and 36 encounters (6.9%) involved a discharge from the ED. The most common discharge disposition was to the street, shelter, or home (368 encounters [70.9%]), followed by LTACH, SNF, or acute physical rehabilitation (137 [26.4%]). The most frequent discharge day was Friday (210 encounters [40.5%]), followed by Saturday (98 [18.9%]), and the median (IQR) length of hospital stay was 6 (3-13) days. Naloxone was prescribed in 90.2% of encounters (468).^41,42^

**Table 2.** Encounter and Methadone Characteristics by 72-Hour OTP Linkage Status

Characteristic	OTP linkage, No. (%)			
	Yes (n = 231)	No (n = 67)	Unknown (n = 221)	Total (n = 519)
**Hospital and emergency department encounter characteristics**				
Sex				
Female	112 (48.5)	29 (43.3)	98 (44.3)	239 (46.1)
Male	119 (51.5)	38 (56.7)	123 (55.7)	280 (53.9)
Age, mean (SD), y	46 (12)	46 (13)	47 (12)	47 (12)
Age, median (IQR), y	45 (36-57)	44 (35-55)	47 (37-57)	46 (36-56)
Race				
Black	69 (29.9)	23 (34.3)	75 (34.0)	167 (32.2)
White	155 (67.1)	43 (64.2)	139 (63.0)	337 (64.9)
Multiracial	0	0	1 (0.5)	1 (0.2)
Other^a^	7 (3.0)	1 (1.5)	6 (2.7)	14 (2.7)
Ethnicity				
Hispanic	18 (7.8)	3 (4.5)	10 (4.5)	31 (6.0)
Non-Hispanic	213 (92.2)	64 (95.5)	211 (95.5)	488 (94.0)
Primary insurance				
Medicaid	183 (79.2)	47 (70.2)	171 (77.4)	401 (77.3)
Medicare	33 (14.3)	14 (20.9)	29 (13.1)	76 (14.6)
Private or military	7 (3.0)	2 (3.0)	11 (5.0)	20 (3.9)
Uninsured	8 (3.5)	4 (6.0)	10 (4.5)	22 (4.2)
Hospital state				
Colorado	38 (16.5)	9 (13.4)	27 (12.2)	74 (14.3)
Connecticut	28 (12.1)	4 (6.0)	6 (2.7)	38 (7.3)
Maryland	165 (71.4)	54 (80.6)	188 (85.1)	407 (78.4)
Discharge department				
Emergency department	13 (5.6)	2 (3.0)	21 (10.0)	36 (6.9)
Medicine or a medical subspecialty	157 (68.0)	55 (82.1)	143 (64.7)	355 (68.4)
Obstetrics and gynecology	17 (7.4)	2 (3.0)	7 (3.2)	26 (5.0)
Psychiatry	1 (0.4)	1 (1.5)	12 (5.4)	14 (2.7)
Surgical or surgical subspecialty	43 (18.6)	7 (10.5)	38 (17.2)	88 (17.0)
Discharge disposition				
Leaving prior to treatment completion or discharged to jail^b^	5 (2.2)	1 (1.5)	8 (3.6)	14 (2.7)
LTACH, or SNF, or acute rehabilitation facility^c^	82 (35.5)	14 (20.9)	41 (18.6)	137 (26.4)
Street, shelter, or home	144 (62.3)	52 (77.6)	172 (77.8)	368 (70.9)
Day of discharge				
Monday	16 (6.9)	10 (14.9)	21 (9.5)	47 (9.1)
Tuesday	17 (7.4)	7 (10.5)	13 (5.9)	37 (7.1)
Wednesday	25 (10.8)	6 (9.0)	10 (4.5)	41 (7.9)
Thursday	28 (12.1)	7 (10.5)	22 (10.0)	57 (11.0)
Friday	93 (40.3)	24 (35.8)	93 (42.1)	210 (40.5)
Saturday	42 (18.2)	9 (13.4)	47 (21.3)	98 (18.9)
Sunday	10 (4.3)	4 (6.0)	15 (6.8)	29 (5.6)
Naloxone ordered at discharge				
No	23 (10.0)	1 (1.5)	27 (12.2)	51 (9.8)
Yes	208 (90.0)	66 (98.5)	194 (87.8)	468 (90.2)
Hospital length of stay, mean (SD), d	10 (11)	10 (11)	9 (12)	10 (12)
Hospital length of stay, median (IQR), d	7 (3-13)	5 (3-15)	5 (3-11)	6 (3-13)
Encounter involving alcohol				
No	168 (72.7)	56 (83.6)	169 (76.5)	393 (75.7)
Yes	63 (27.3)	11 (16.4)	52 (23.5)	126 (24.3)
Encounter involving stimulants (methamphetamine and/or cocaine)				
No	106 (45.9)	19 (28.4)	98 (44.3)	223 (43.0)
Yes	125 (54.1)	48 (71.6)	123 (55.7)	296 (57.0)
Encounter involving benzodiazepines				
No	182 (78.8)	55 (82.1)	173 (78.3)	410 (79.0)
Yes	49 (21.2)	12 (17.9)	48 (21.7)	109 (21.0)
Encounter involving drug poisoning/overdose				
No	215 (93.1)	61 (91.0)	208 (94.1)	484 (93.3)
Yes	16 (6.9)	6 (9.0)	13 (5.9)	35 (6.7)
Encounter involving pregnancy				
No	212 (91.8)	64 (95.5)	212 (95.9)	488 (94.0)
Yes	19 (8.2)	3 (4.5)	9 (4.1)	31 (6.0)
Encounter involving skin/soft tissue infection, musculoskeletal infection, endocarditis, bacteremia				
No	140 (60.6)	33 (49.2)	149 (67.4)	322 (62.0)
Yes	91 (39.4)	34 (50.8)	72 (32.6)	197 (38.0)
Encounter involving trauma/burn				
No	208 (90.0)	66 (98.5)	186 (84.2)	460 (88.6)
Yes	23 (10.0)	1 (1.5)	35 (15.8)	59 (11.4)
Encounter involving a psychiatric condition				
No	140 (60.6)	45 (67.2)	139 (62.9)	324 (62.4)
Yes	91 (39.4)	22 (32.8)	82 (37.1)	195 (37.6)
Encounter involving a psychiatric condition				
ADHD	1 (0.4)	0	0	1 (0.2)
Anxiety or depressive disorder	53 (22.9)	18 (26.9)	55 (24.9)	126 (24.3)
Bipolar disorder	18 (7.8)	1 (1.5)	14 (6.3)	33 (6.7)
PTSD	1 (0.4)	0	1 (0.5)	2 (0.4)
Other psychotic conditions	14 (6.1)	3 (4.5)	7 (3.2)	24 (4.6)
Schizophrenia	3 (1.3)	0	3 (1.4)	6 (1.2)
**Methadone-related characteristics**				
Enrolled in an OTP at admission				
No	104 (45.0)	45 (67.2)	101 (45.7)	250 (48.2)
Yes	127 (55.0)	22 (32.8)	120 (54.3)	269 (51.8)
No. of methadone doses dispensed, mean (SD)	2.15 (0.84)	2.04 (0.91)	2.05 (0.82)	2.09 (0.84)
No. of methadone doses dispensed, median (IQR)	2 (1-3)	2 (1-3)	2 (1-3)	2 (1-3)
Methadone dose, mean (SD), mg	82.53 (33.03)	65.82 (24.64)	74.74 (33.71)	77.09 (32.83)
Methadone dose, median (IQR), mg	80 (60-100)	60 (50-80)	70 (50-100)	70 (55-100)
Reason for dispensing methadone				
Holiday	7 (3.0)	3 (4.5)	12 (5.4)	22 (4.2)
LTACH, SNF, or acute rehabilitation facility	50 (21.7)	4 (6.0)	31 (14.0)	85 (16.4)
Other^d^	26 (11.3)	17 (25.4)	27 (12.2)	70 (13.5)
Weekend	148 (64.1)	43 (64.9)	151 (68.3)	342 (65.9)

Abbreviations: ADHD, attention deficit hyperactivity disorder; LTACH, long-term acute care hospital; OTP, opioid treatment program; PTSD, posttraumatic stress disorder;

SNF, skilled nursing facility.

^a^ Other category included American Indian or Alaska Native, Asian, and Pacific Islander or Native Hawaiian and was created due to small numbers in each group.

^b^ Three patients were discharged to jail, 11 left prior to treatment completion.

^c^ Two patients were discharged to an acute physical rehabilitation facility, 2 patients were discharged to an LTACH.

^d^ Most frequently due to travel distance.

One hundred twenty-six encounters (24.4%) involved co-use of opioids and alcohol, 296 (57.4%) involved co-use of opioids and stimulants (either cocaine and/or methamphetamine), 109 (21.1%) involved co-use of opioids and benzodiazepines, and 35 (6.7%) involved drug overdose or poisoning. Six percent of encounters (31) involved pregnancy; 38.0% (197) involved infection; 11.4% (59) involved burn or trauma; and 37.6% (195) involved psychiatric conditions, with depression and/or anxiety as the leading psychiatric condition listed (126 [24.3%]).

### Methadone-Related Characteristics

A total of 269 encounters (51.8%) involved patients who were enrolled in an OTP at the time of their acute care encounter. The median (IQR) number of methadone doses dispensed per encounter was 2 (1-3) and the median (IQR) methadone dose dispensed was 70 (55-100) mg. The primary reasons for giving patients take-home methadone were weekend discharge (342 [65.9%]) and discharge to a SNF, LTACH, or acute physical rehabilitation (85 [16.4%]). In all, 231 encounters (44.5%) had a complete 72-hour hospital to OTP linkage status, 67 (12.9%) did not have a complete 72-hour OTP linkage status, and 221 (42.6%) had an unknown 72-hour OTP linkage status.

### Associations Between 72-Hour OTP Linkage and Variables of Interest

Variables associated with a 72-hour hospital to OTP linkage status of yes included discharge disposition to an LTACH, SNF, or acute physical rehabilitation (vs discharge disposition to the street, shelter, or home) (odds ratio [OR], 2.12; 95% CI, 1.12-4.21; *P* = .02) (Table 3). OTP enrollment at the time of the acute care encounter was associated with both an OTP linkage status of yes (OR, 2.63; 95% CI 1.48-4.79; *P* < .001) and an OTP linkage status of unknown (OR, 2.41; 95% CI, 1.35-4.40; *P* = .003). Higher methadone doses in 10mg increments were associated with both OTP linkage status yes (OR, 1.20; 95% CI, 1.09-1.33; *P* < .001) and OTP linkage status unknown (OR, 1.12; 95% CI, 1.01-1.24; *P* = .02). Encounters involving stimulants were inversely associated both with 72-hour OTP linkage status yes (OR, 0.48; 95% CI, 0.26-0.87; *P* = .01) and OTP linkage status unknown (OR, 0.51; 95% CI, 0.28-0.93; *P* = .03). Leaving prior to treatment completion or discharged to jail, encounters involving pregnancy, encounters involving a psychiatric condition, and hospital length of stay were not associated with 72-hour linkage. In the multinomial regression models, sex, age, race, and ethnicity were not associated with 72-hour linkage.

**Table 3.** Odds of 72-Hour Hospital-to-OTP Linkage by Variable of Interest^a^^,^^b^

**Variable and hospital to OTP linkage outcome **	**Odds ratio (95% CI)**	***P* value**
Discharge disposition		
Street, shelter, or home	1 [Reference]	NA
Left prior to treatment completion or discharged to jail		
Unknown	2.48 (0.44-46.67)	.35
Yes	1.85 (0.29-36.07)	.55
No	1 [Reference]	NA
LTACH, SNF, or acute rehabilitation facility		
Unknown	0.86 (0.44-1.76)	.68
Yes	2.12 (1.12-4.21)	.02
No	1 [Reference]	NA
Enrolled in an OTP at hospital admission (yes)		
Unknown	2.41 (1.35-4.40)	.003
Yes	2.63 (1.48-4.79)	<.001
No	1 [Reference]	NA
Hospitalization involving stimulants (yes)		
Unknown	0.51 (0.28-0.93)	.03
Yes	0.48 (0.26-0.87)	.01
No	1 [Reference]	NA
Hospitalization involving pregnancy (yes)		
Unknown	1.04 (0.27-5.10)	.96
Yes	2.11 (0.62-9.75)	.25
No	1 [Reference]	NA
Hospitalization involving a psychiatric condition (yes)		
Unknown	1.21 (0.68-2.20)	.52
Yes	1.35 (0.77-2.44)	.30
No	1 [Reference]	NA
Hospital length of stay (per 1 d increase)		
Unknown	0.99 (0.97-1.02)	.64
Yes	1.00 (0.98-1.03)	.91
No	1 [Reference]	NA
Methadone dose (increasing 10-mg increments)		
Unknown	1.12 (1.01-1.24)	.02
Yes	1.20 (1.09-1.33)	<.001
No	1 [Reference]	NA

Abbreviations: LTACH, long-term acute care hospital; OTP, opioid treatment program; SNF, skilled nursing facility.

^a^ Hospital to OTP linkage: yes, 231 encounters; unknown, 221 encounters; no, 67 encounters.

^b^ Inclusion of age (increasing years), race (Black, White, or other [other category created due to small numbers]), sex (male or female), and ethnicity (Hispanic or non-Hispanic) in each multinomial regression model did not yield associations between the variable of interest and odds of hospital to OTP linkage within 72 hours after discharge, suggesting that these covariates were not associated with the outcome in the final models.

## Discussion

Despite decades of evidence demonstrating the effectiveness of methadone managing for OUD,^7,8,43^ US federal oversight and regulations have historically limited methadone access, especially during health care transitions.^44,45^ Take-home methadone processes offer new opportunities to support patients during acute care transitions while avoiding a period of opioid withdrawal, especially when OTPs may be closed or have limited hours. This is important because untreated OUD is associated with increased risk of death,^46^ whereas the use of medications for OUD has been reported to have protective benefits against acute care utilization,^6^ overdose death,^6^ and all-cause mortality.^3,5,21^

To our knowledge, the current study is the first to describe outcomes related to take-home methadone in response to a change in the federal 72-hour rule, allowing for up to a 3-day supply of methadone to be dispensed by the hospital at one time. Studies conducted before the federal regulation change that involved hospitalized patients with OUD reported hospital to OTP linkage rates (without dispensing of discharge methadone doses) that ranged from 40% to 76%.^35,47,48^ Before the federal regulation change, an emergency department^15^ and an outpatient clinic^26^ each implemented innovative methods to dispense a methadone “guest” dose to patients with OUD to facilitate OTP linkage under the original 72-hour rule. These studies reported OTP linkage rates that ranged from 56% to 87%.^15,26^ In contrast to our study, these studies reported linkage completion at partnering and affiliated OTPs and did not report on linkage completion at nonpartner or nonaffiliated OTPs.^35,47,48^ Our 44% linkage rate was lower than those previously reported and may underrepresent the completed hospital to OTP linkage rate because we did not exclude patients who preferred to follow up at nonaffiliated and nonpartner OTPs.^15,35,47^ In our study, we had no reason to assume that patients with an unknown linkage status were less likely to complete a 72-hour hospital to OTP linkage compared with patients who preferred to follow up at an affiliated or partnering OTP. Our clinical experience suggests that patients prefer to follow up at OTPs that are convenient or familiar to them, and this includes nonpartner and nonaffiliated OTPs, particularly if they live further away from the admitting hospital. Future work should study the association of acute care transition interventions with linkage to all OTPs to provide a greater understanding of their association with completed OTP linkage.

We found that patients discharged to LTACH, SNF, or acute physical rehabilitation facilities were more likely to complete a 72-hour hospital-to-OTP linkage. LTACHs, which are designated as hospitals, may legally dispense methadone to prevent opioid withdrawal, even when a patient is not enrolled in an OTP.^14^ This is also true for acute rehabilitation facilities located within hospitals.^14^ If a patient is continued on methadone in an LTACH or an acute rehabilitation facility located in a hospital for prevention of withdrawal (ie, they are not enrolled in an OTP), the patient must be enrolled in an OTP after discharge to continue methadone for OUD treatment.

In contrast to LTACHs, SNFs are not designated as hospitals. Federal law prohibits SNFs from continuing methadone beyond 72 hours if the patient is not enrolled in an OTP. There are 3 exceptions: (1) the SNF is accredited and certified as an OTP, (2) the SNF has an on-site medication unit under an OTP, or (3) the patient is already enrolled in an OTP and is receiving methadone for OUD. In the latter scenario, the OTP will deliver the patient’s methadone doses to the SNF for administration under a “guest” dosing arrangement. In our study, 3 sites (UCH, JHH, and JHBMC) facilitated in-hospital OTP enrollments to ensure methadone continuation at the SNF.^47,49,50^ The take-home methadone doses allowed for time to arrange methadone deliveries to the SNF, especially on weekends and holidays when OTPs had limited hours or were closed, facilitating hospital discharges and thereby reducing the length of hospital stay and health care costs for medically stable patients.^51^ Three sites (JHH, JHBMC, and YNHH) identified SNFs that agreed to accommodate patients on methadone. One site had preexisting agreements between OTPs and SNFs, a practice supported by Connecticut’s Department of Health initiatives.^52^ None of the sites had a memorandum of understanding between SNFs and OTPs to facilitate this process. Finally, if a patient was not enrolled in an OTP prior to SNF transfer, a 3-day supply of methadone could theoretically provide time to arrange a telehealth OTP intake while the patient was at the SNF, in accordance with the updated Substance Abuse and Mental Health Services Administration’s Federal Guidelines for Opioid Treatment Programs Final Rule.^11^ The availability of take-home methadone and partnerships between SNFs and OTPs facilitated care coordination to ensure patients continued receiving methadone following care transitions, an often tenuous time for people with OUD.^18^

Increasing methadone doses in 10-mg increments were associated with hospital to OTP linkage status of yes and unknown. This finding is consistent with previously reported research demonstrating greater OTP engagement and retention with higher vs lower methadone doses^29,30,31^ and supports in-hospital methadone titration to comfort.

Hospitalizations involving stimulants were inversely associated with OTP linkage. This finding is consistent with studies demonstrating that people who co-use opioids and stimulants are less likely to engage with, or remain engaged in, substance treatment.^34,35^ Expansion of in-hospital contingency management programs for people using stimulants may improve substance treatment engagement.^53,54,55^

### Limitations

This study has several limitations. We analyzed data at the encounter level instead of the patient level because we aimed to study outcomes related to an encounter-level intervention, take-home methadone. Limiting data to the patient level would mean eliminating data on patients with more than 1 hospitalization in which take-home methadone was dispensed. Twelve percent of the encounters represented repeated acute care encounters by individual patients. The models that incorporated more than 1 encounter per patient were unstable, likely due to the small number of within-patient observations, which limited our ability to estimate random effects. Thus, we did not include these data in the regression models. By treating observations as independent, our final model may have underestimated SEs and overstated statistical significance, potentially biasing association estimates. We were unable to capture factors potentially associated with OTP linkage, including housing status and access to transportation or identification, which may have confounded our results.^16,56,57^ All study sites had an ACS that supported take-home methadone implementation and clinical management, which may limit generalizability to sites without an ACS; however, a clinician or pharmacist could also champion these processes. ED encounters were minimally represented because only 1 site offered take-home methadone to this population. Lastly, our study findings were limited to academic hospitals located in communities with OTPs and are not generalizable to locations lacking OTP access.^58,59^

## Conclusions

In this multisite cohort study of hospitals with addiction support, preexisting relationships between hospitals, postacute care facilities, and OTPs, and provision of take-home methadone, likely facilitated hospital to OTP linkage within 72 hours of hospital discharge. Expansion of these relationships and processes could facilitate OUD treatment engagement during tenuous care transitions.

## Notes

Supplement.
Data Sharing Statement
