# Supplementary material for: Leveraging the 72-Hour Rule Change to Support Transition From Hospital to Opioid Treatment Program
Source: JAMA Netw Open. 2025 Nov 21;8(11):e2544996. doi: 10.1001/jamanetworkopen.2025.44996 (PMC12639479; doi:10.1001/jamanetworkopen.2025.44996)
Supplement: Supplement. — Data Sharing Statement [file jamanetwopen-e2544996-s001.pdf]

Data Sharing Statement

Calcaterra. Leveraging the 72-Hour Rule Change to Support Transition From Hospital to Opioid Treatment Program. *JAMA Netw Open*. Published November 21, 2025. doi:10.1001/jamanetworkopen.2025.44996

Data
Data available: No
